# Bayesian mixture analysis for metagenomic community profiling

**DOI:** 10.1093/bioinformatics/btv317

**Published:** 2015-05-21

**Authors:** Sofia Morfopoulou, Vincent Plagnol

**Affiliations:** UCL Genetics Institute, University College London, London WC1E 6BT, UK

## Abstract

**Motivation:** Deep sequencing of clinical samples is now an established tool for the detection of infectious pathogens, with direct medical applications. The large amount of data generated produces an opportunity to detect species even at very low levels, provided that computational tools can effectively profile the relevant metagenomic communities. Data interpretation is complicated by the fact that short sequencing reads can match multiple organisms and by the lack of completeness of existing databases, in particular for viral pathogens. Here we present metaMix, a Bayesian mixture model framework for resolving complex metagenomic mixtures. We show that the use of parallel Monte Carlo Markov chains for the exploration of the species space enables the identification of the set of species most likely to contribute to the mixture.

**Results:** We demonstrate the greater accuracy of metaMix compared with relevant methods, particularly for profiling complex communities consisting of several related species. We designed metaMix specifically for the analysis of deep transcriptome sequencing datasets, with a focus on viral pathogen detection; however, the principles are generally applicable to all types of metagenomic mixtures.

**Availability and implementation:** metaMix is implemented as a user friendly R package, freely available on CRAN: http://cran.r-project.org/web/packages/metaMix

**Contact:** sofia.morfopoulou.10@ucl.ac.uk

**Supplementary information:** Supplementary data are available at *Bionformatics* online.

## 1 Introduction

Metagenomics can be defined as the study of DNA sequences from environmental or community samples, while metatranscriptomics is the analysis of RNA sequence data from such samples. The scope of metagenomics/metatranscriptomics is broad and includes the analysis of a diverse set of samples such as gut microbiome (Minot *et al.*, 2011; Qin *etal.*, 2010), environmental (Mizuno *etal.*, 2013) or clinical (McMullan *etal.*, 2012; Negredo *etal.*, 2011; Willner *etal.*, 2009) samples. Among these applications, the discovery of viral pathogens is clearly relevant for clinical practice (Chiu, 2013; Fancello *etal.*, 2012). The traditional process of characterizing a virus through potentially difficult and time consuming culture techniques is being revolutionized by advances in high throughput sequencing. Potential benefits of sequence driven methodologies include a more rapid turnaround time (Quail *etal.*, 2012), combined with a largely unbiased approach in species detection, including the opportunity for unexpected discoveries.

The analysis of shotgun sequencing data from metagenomic mixtures raises complex computational challenges. Part of the difficulty stems from the read length limitation of existing deep DNA-sequencing technologies, an issue compounded by the extensive level of homology across viral and bacterial species. Another complication is the divergence of the microbial DNA sequences from the publicly available references. As a consequence, the assignment of a sequencing read to a database organism is often unclear. Last, the number of reads originating from a disease causing pathogen can be low (Barzon *etal.*, 2013). The pathogen contribution to the mixture depends on the biological context, the timing of sample extraction and the type of pathogen considered. Therefore, highly sensitive computational approaches are required.

A first analytical problem is read classification that is the assignment of a given sequencing read to a species. Several tools have been developed and these belong to two broadly defined classes: composition-based and similarity-based approaches. The read classification based on sequence composition relies on the intrinsic features of the reads. Methods include PhyloPythia (McHardy *etal.*, 2007) and Phymm (Brady and Salzberg, 2009). These tend to focus on major classes in a dataset and may not perform well on low-abundance populations (Kunin *etal.*, 2008). Additionally, results are usually reliable for longer reads only (Dröge and McHardy, 2012).

Similarity-based methods, using homology search algorithms such as BLAST (Altschul *etal.*, 1990), are considered the most sensitive methods for read classification (Brady and Salzberg, 2009). One of the most popular tools using the output of a similarity search algorithm is MEGAN (Huson *etal.*, 2007). MEGAN addresses ambiguous matches by assigning reads that have multiple possible assignments to several species, to the taxonomic group containing all these species, or else their lowest common ancestor (LCA). This approach is accurate on a higher taxonomic level. However, it is lacking a formal solution to resolving ambiguous matches.

A weakness of the similarity-based methods is that a long tail of species, each supported only by few reads can appear in the results. This results from the classification being decided one read at a time, in contrast to considering all reads simultaneously. Hybrid methods combining composition and similarity information such as PhymmBL (Brady and Salzberg, 2009) and RITA (MacDonald *etal.*, 2012) also work read by read.

Methods focused on the statistical inference of the set of present species as well as the estimation of their relative proportions, incorporate knowledge from all reads to assign each individual read to a species. From a statistical standpoint, this identification and quantification question can be thought of as an application of mixture models. These ideas have been applied in the metagenomics context in frequentist [GRAMMy (Xia *etal.*, 2011)] and Bayesian [Pathoscope (Francis *etal.*, 2013)] settings. GRAMMy formulates the problem as a finite mixture model, using the Expectation-Maximization (EM) algorithm to estimate the relative genome abundances. Pathoscope refines this process by penalizing reads with ambiguous matches in the presence of reads with unique matches and enforcing parsimony within a Bayesian context.

Fitting a mixture model is useful for the species relative abundance estimation, as well as the read to species assignment. A related but distinct question concerns the set of species which should be included in the mixture model. This question is closely related to the biological question of asking what species are present in the mixture. Including all species flagged as potential matches by the read classification can introduce a large number of species, often in the low thousands. Mixture models will then identify a large number of species at low levels. This interpretation is appropriate in some applications. In many other cases, the expectation is that the underlying species set should be parsimonious and that some divergence with database species or sequencing errors can explain a large fraction of the non matching reads.

Hence, a better statistical formulation of the community profiling problem is the exploration of the candidate organisms state-space. In this context, non nested models can be compared based on their marginal likelihood. Within this Bayesian framework, readily interpretable probabilities, such as the posterior probabilities of species sets can be used to quantify the support for a species in the mixture. Finally, more complex hypotheses testing for example the number of viral species or the joint presence of two distinct organisms can be investigated.

The main challenge behind such a formulation is computational. Even with a relatively small number of species to consider, the number of subsets of this space that could explain the mixture grows exponentially. Efficient computational strategies are required to make this problem tractable. Here we show that this inference can be achieved for modern scale metagenomics datasets. Our strategy is based on parallel tempering, a Monte Carlo Markov Chain (MCMC) technique, using parallel computing to speed up the inference. We implemented these ideas in a user friendly R package called metaMix. metaMix produces posterior probabilities for various models as well as the relative abundances under each model. We demonstrate its potential using datasets from clinical samples as well as benchmark metagenomic datasets.

## 2 Methods

### 2.1 Bioinformatics preprocessing

Prior to running the mixture model for metagenomic profiling, several steps are required to process the short read sequence data. The pipeline uses publicly available bioinformatics tools for each preprocessing step.

The first step is the removal of clonal reads using an in house C++ script. We then use PRINSEQ (Schmieder and Edwards, 2011) for read-based quality control, removing low quality and complexity reads and performing 3′end trimming. For metagenomic analysis of human samples, reads originating from the human host are not relevant for our research question. We therefore remove human host reads, using a two-step approach to limit computation time: initially a short read aligner (novoalign, www.novocraft.com), followed by BLASTn. The next step is only applicable when the focus is on virus discovery using transcriptome reads. We remove ribosomal RNA sequences, using BLASTn against the Silva rRNA database.

The remaining reads are assembled into contigs using the Velvet short read assembler (Zerbino and Birney, 2008). For each contig we record the number of reads required for its assembly, using this information at the stage of species abundance estimation. A Velvet tuning parameter is the user defined *k*-mer length that specifies the extent of overlap required to assemble read pairs. Metagenomic assembly is not a straightforward task, as short k-mers work best with the low abundance organisms, while long k-mers with the highly abundance ones. The shorter the k-mer the greater the chance of spurious overlaps, hence we choose relatively high *k*-mer length, in order to avoid chimeric contigs.

For each contig and unassembled read we record the potentially originating species, using the nucleotide to protein homology matching tool BLASTx. We use BLASTx due to the higher level of conservation expected at the protein level compared with nucleotides. This choice is guided by our focus in viral pathogens—viruses having high genetic diversity and divergence (Fancello *etal.*, 2012). If taxonomic information is not included in the BLAST output, we obtain it by using the NCBI taxonomy files that map proteins to taxons. For simplicity we subsequently drop the protein information and only keep a record of mismatches between the read and the species. If a read matches multiple proteins from the same species, we keep only the best match. This step generates a sparse similarity matrix between the read sequences and the protein sequences, with species as columns, reads and contigs as rows.

The statistical method described in the remainder of this section considers the competing models that could accommodate our observed data i.e. the BLASTx results and compares them. The different models represent different sets of species being present in the sample. The method works on two levels of inference: in the first instance we assume a set of species to be present in the sample and we estimate this model’s parameters given the data. The other level of inference is the model comparison so as to assess the more plausible model. The process is iterated in order to explore the model state space.

### 2.2 Model specification assuming a fixed set of species

Assuming a given set of *K* species from which the reads can originate, the metagenomic problem can be summarized as a mixture problem, for which the assignment of the sequencing reads to species is unknown and must be determined. The data consist of *N* sequencing reads $X=(x_{1},\ldots ,x_{N})$, and for a given read *x_i_* the likelihood is written as:

(1) $$p(x_{i}|w,K)=p(x_{i}|w)=\sum_{j=1}^{\text{K}}w_{j}f_{j}(x_{i})$$

where $w=(w_{1},...,w_{K})$ represent the proportion of each of the *K* species in the mixture. These mixture weights are constrained such that $0\leq w_{j}\leq 1$ and $\sum_{j}wj=1$. In practice, we also add a category (species *K* + 1) which we refer to as the ‘unknown’ category, and captures the fact that some reads cannot be assigned to any species.

Additionally $f_{j}(x_{i})$ = $P(x_{i}|x_{i}\;\text{from}\;\text{species}\;j)$ = *p*_ij_ is the probability of observing the read *x_i_* conditional on the assumption that it originated from species *j.* We model this probability using the number of mismatches *m* between the translated read sequence and the reference sequence and a Poisson distribution with parameter *λ* for that number of mismatches $p_{\text{ij}}=\text{Pois}(m;\lambda )/l_{\text{g}}$, where *l*_g_ is the length of the reference genome, when short reads are matched to a nucleotide database. For nucleotide matching, *l*_g_ has a large impact on the probability computation. However, when matching against protein databases, the more limited heterogeneity of protein lengths results in a much smaller impact of the length parameter. In addition, incomplete annotation can potentially make the inclusion of protein length problematic for the *p*_ij_ computation. Consequently, for protein matched sequences, we simply defined our *p_ij_* as: $p_{\text{ij}}=\text{Pois}(m;\lambda )$.

Therefore for a given set of *K* species, the *p*_ij_ probabilities are regarded as known and the mixture weights must be estimated. Combining the above we see that when we know the set of species *K*, the mixture distribution gives the probability of observing read *x_i_*: $\sum_{j=1}^{\text{K}}W_{j}p_{\text{ij}}$, i.e Equation (1).

We therefore write the likelihood of the dataset ***X*** as a sum of *K*^n^ terms:

(2) $$P(X|w)=\prod_{i=1}^{\text{n}}\left[\sum_{j=1}^{\text{K}}w_{j}p_{\text{ij}}\right]$$

### 2.3 Estimation of mixture weights

Assuming a fixed set of species, the posterior probability distribution of the weights ***w*** given the read data *X* is:

(3) $$P(w|X)=\frac{P(X|w)\pi (w)}{P(X)}\overset{\left(2\right)}{=}\frac{\prod_{i}\left[\sum_{i}w_{j}p_{\text{ij}}\right]\pi (w)}{\int \prod_{i}\left[\sum_{i}w_{j}p_{\text{ij}}\right]\pi (w)dw}$$

A practical prior for the mixing parameters ***w*** is the Dirichlet distribution owing to its conjugate status to the multinomial distribution. Despite the use of conjugate priors, the probabilistic assignment of reads to species involves the expansion of the likelihood into *K*^n^ terms which is computationally infeasible through direct computation. An efficient estimation can be performed by the introduction of unobserved latent variables that code for the read assignments. In this framework, either the Gibbs sampler (Diebolt and Robert, 1994; Marin *et al.*, 2005) a MCM technique, or the EM (Dempster and Laird, 1977) algorithm can be used to estimate the mixture weights ***w***. EM returns a point estimate for ***w*** while the Gibbs sampler the distribution of ***w*** (Supplementary Methods). Both methods were implemented and provided comparable results.

### 2.4 Marginal likelihood estimation

Each combination of species corresponds to a finite mixture model for which the marginal likelihood can be estimated. Marginal likelihood comparison has a central role in comparing different models $\left\{M_{1},\ldots ,M_{m}\right\}$. To compute the marginal likelihood $P(X|M_{\text{k}})$ for the mixture model *M*_k_ one has to average over the parameters with respect to the prior distribution $\pi (\theta_{k}|M_{\text{k}})$, where $\theta_{\text{k}}$ are the model parameters:

(4) $$P(X|M_{\text{k}})=\int_{\theta_{\text{k}}}P(X|\theta_{\text{k}},M_{\text{k}})\pi (\theta_{\text{k}}|M_{\text{k}})d\theta_{\text{k}}$$

The posterior probability of the model *M*_k_ is:

(5) $$P(M_{\text{k}}|X)\propto P(X|M_{\text{k}})P(M_{\text{k}})$$

where $P(M_{\text{k}})$ is the prior belief we hold for each model. The prior can be specified depending on the context but the basis of our interpretation is that parsimonious models with a limited number of species are more likely. Thus, in this Bayesian framework, our default prior uses a penalty limiting the number of species in the model, i.e $P(M_{\text{k}})={\text{penalty}}^{(\text{number of species in }M_{\text{k}}\text{)}}$. We approximate this penalty factor based on a user-defined parameter r that represents the species read support required by the user to believe in the presence of this species. We compute the logarithimic penalty value as the log-likelihood difference between two models: model $M_{\text{unknown}}$ which is our starting point when we have no knowledge about which species are present and therefore all *N* reads come from the ‘unknown’ category ($p_{\text{ij}}=10^{-6}$) and model $M_{\text{r}}$ where r reads have a perfect match to a species (*p*_ij_ = 1) and the remaining N−r reads belong to the ‘unknown’ category: $\log\text{penalty}=\log P(M_{\text{unknown}}|X)-\log P(M_{\text{r}}|X)$. For DNA sequence analysis, the *p*_ij_ probabilities for the r reads originating from this unspecified species are approximated by 1/(median genome length in the reference database). This read support parameter reflects, in our probabilistic framework, the number of unique reads required to support the hypothesis that a species is present.

From now on, when we refer to the marginal likelihood, we mean the marginal likelihood for a specific model and we forego conditioning on the model *M*_k_ in the notation. Additionally, in our mixture model *p_ij_* are always regarded as known, therefore the model parameters $\theta_{\text{k}}$ are the mixture weights ***w***. Hence (4) becomes:

(6) $$P(X)=\int_{\text{w}}P(X|w)\pi (w)dw\overset{\left(2\right)}{=}\int_{\text{w}}\prod_{\text{i}}\left[\sum_{\text{i}}w_{\text{j}}p_{\text{ij}}\right]\pi (w)dw$$

Approximating the marginal likelihood is a task both difficult and time-consuming. We chose the Defensive Importance Sampling technique (Hesterberg, 1995) for the relative simple implementation compared with other approaches (Supplementary Methods for details of implementation). This is crucial as we perform this approximation numerous times, for every species combination we consider.

However the goal of this work is to deliver results in a clinical setting within an actionable time-frame. We wish to speed up the computation without compromising the accuracy and the sensitivity of the results. For that reason, we use a point estimate of the marginal likelihood by means of the EM algorithm. The different approaches were used on the benchmark dataset. The resulting taxonomic assignment as well as the species relative abundance estimates were similar between them, with the EM approach resulting in a 13-fold speed increase (Supplementary Methods).

### 2.5 Model comparison: exploring the set of present species

We use a MCMC to explore the set of present species of size $2^{\text{S}}-1$, where *S* is the total number of potential species. In practice we observe that *S* can be > 1000. The MCMC must explore the state-space in a clinically useful timespan. Therefore we reduce the size of the state-space, by decreasing the number of *S* species to the low hundreds. We achieve this by fitting a mixture model with *S* categories, considering all potential species simultaneously. Post fitting, we retain only the species categories that are not empty, that is categories that have at least one read assigned to them.

Let us assume that at step *t*, we deal with a set of species that corresponds to the mixture model *M*_k_. At the next step (t+1), we either add or remove a species and the new set corresponds to the mixture model *M*_l_. The step proposing the model *M*_l_ is accepted with probability:

(7) $$A(M_{\text{k}}\to M_{\text{l}})=\text{min}\left\{1,\frac{P{\left(X|M_{l}\right)}^{\left(t+1\right)}P(M_{\text{l}})}{P{\left(X|M_{\text{k}}\right)}^{\left(\text{t}\right)}P(M_{\text{k}})}\frac{q(M_{\text{l}}\to M_{\text{k}})}{q(M_{\text{k}}\to M_{\text{l}})}\right\}$$

where $q(M_{\text{l}}\to M_{\text{k}})$ is the probability of transitioning from model *M*_l_ to model *M*_k_. In other words, this is the probability of adding or removing the species to the *M*_k_ set of species that took us to the *M*_l_ set of species.

If the step is accepted, then the chain moves to the new proposed state *M*_l_. Otherwise if not accepted, the chain’s current state becomes the previous state of the chain, i.e the set of species remains unchanged.

metaMix outputs log-likelihood traceplots so that the user can visually inspect the mixing and the convergence of the chain. The default setting is to discard the first 20% of the iterations as burn-in. We concentrate on the rest to study the distribution over the model choices and perform model averaging (Hoeting *etal.*, 1999), incorporating model uncertainty. This framework allows to answer a broad range of questions. For example, what species have probability *p* or greater being included in the set of present species? Finally, metaMix also outputs Bayes Factors to quantify the evidence in favour of each species:

$$\log_{10}\text{BF}=\log_{10}\frac{P(X|M_{\text{species present}})}{P(X|M_{\text{species absent}})}$$.

#### 2.6 Optimized implementation: parallel tempering

**Fig. 1.**
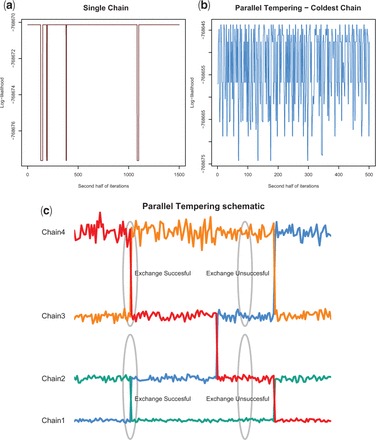
**a.** Log-likelihood trace plot for single chain MCMC and **b.** for PT chain at temperature *T* = 1. **c.** Schematic of parallel tempering. Exchanges are attempted between chains of neighboring temperatures, where Chain1 at $T_{1}=1,\;T_{1}<T_{2}<T_{3}<T_{4}$

We observed that simple MCMC does not efficiently explore the complex model state space, as evidenced by the poor mixing of the chain (Fig. 1). In order to overcome this and take advantage of parallel computing, we run multiple chains and allow exchange moves between them. This method is called parallel tempering MCMC (Earl and Deem, 2005). 

Within the parallel setting, each chain simulates from the posterior distribution $P(M_{\text{k}}|X)$ = $g(M_{\text{k}})$ raised to a temperature t∈(0,1], where model *M_k_* comes from a collection of models $\left\{M_{1},\ldots ,M_{m}\right\}$ and represents a set of species being present. The different temperature levels result in tempered versions of the posterior distribution $P{\left(M_{\text{k}}|X\right)}^{t=1/T}$. When *T* = 1 the draws are from the posterior distribution. On the other hand, at higher temperatures the posterior spreads out its mass and becomes flatter. In practice that means that distributions at higher temperatures are easily sampled, improving the mixing. We are interested in studying the original posterior distribution with *T* = 1.

We implemented two types of moves. The first is the mutation step, which simply is the within chain move we described in the previous section. This is accepted with probability given by (7). The other is the exchange step, a between chains move. This Metropolis-Hastings move proposes to swap the value of two chains *k* and *k* + 1, adjacent in terms of *T*, with respective temperatures 1/*T*_1_ and 1/*T*_2_ where $T_{1}<T_{2}$. Suppose that the values of the two chains are *M*_k_ and $M_{k+1}$, respectively, corresponding to two different sets of species. The move is accepted with probability (Jasra *etal.*, 2007):

(8) $$A=\text{min}\left\{1,\frac{g_{\text{k}}(M_{k+1})}{g_{\text{k}}(M_{\text{k}})}\frac{g_{k+1}(M_{\text{k}})}{g_{k+1}(M_{k+1})}\right\}$$

Because $g_{\text{k}}(M_{\text{k}})$ = $P{\left(M_{\text{k}}|X\right)}^{1/T_{1}}$ and $g_{k+1}(M_{k+1})$ = $P{\left(M_{k+1}|X\right)}^{1/T_{2}}$, it follows that when $M_{k+1}$ represents a set of species of higher probability than the one *M*_k_ represents, the exchange will always be accepted (Supplementary Methods, Fig. 1). This allows moves between separate modes, ensuring a global exploration of the model state space. Eventually ‘hot’ and ‘cold’ chains will progress towards a global mode.

## 3 Results

We first applied metaMix on a popular benchmark dataset, for which the community composition and the read assignment is known. We then analyzed RNA-Seq datasets from two clinical samples that were generated for diagnostic purposes. We compare our results with the ones produced by MEGAN version 5.3 and Pathoscope 2.0. Both methods are similarity based. This property and more specifically their flexibility to work with BLASTx output, makes them better candidates for viral discovery compared with composition-based methods. From the mixture model methods, we have chosen Pathoscope. We were also interested in comparing our results to the ones by GRAMMy, which was the first similarity-based method to use the idea of the mixture model. However, GRAMMy is designed for nucleotide-nucleotide comparisons (BLASTn), which is suboptimal for viral discovery. GRAMMy also only considers unassembled reads and requires that these are of the same fixed length. For these reasons, GRAMMy was not included in the comparison. Default parameters were used for all methods, unless stated otherwise.

For the metaMix output, we reported organisms with a posterior probability *P* > 0.8 (default). The metaMix read support parameter r, which essentially sets the sensitivity/specificity of the method, has an impact on the number of reported species. A large r value can result in the method merging together strains that are differentiated by fewer reads than r. On the other hand a low r can have the opposite effect, whereby the methods splits a strain into two or more strains, by moving a few reads from one strain to a very similar one with which they have equally good matches.

The user’s choice for this key parameter r should be informed by the biological context. As an example, for the typical human clinical sample where the sample collection might have occurred some time after the infection has taken place, a low value in order to adopt a sensitive approach is reasonable. Hence, for viral identification in human clinical samples, a low and sensitive value (r=10) is a reasonable choice. In a highly complex environmental metagenomic community where there is a plethora of species of similar abundances, the choice becomes less straightforward especially in the case of closely related strains. We set the default value for general community profiling in environmental samples at r=30. We also compare the output of metaMix for different values of this parameter as well as for different posterior probability cutoffs. Supplementary Methods presents a discussion on the metaMix settings as well as practical considerations.

### 3.1 FAMeS datasets—closely related strains

The FAMeS artificial datasets (http://fames.jgi-psf.org/description.html), are mock metagenomic community datasets composed of random reads from 113 isolate microbial genomes. They are a popular choice to use as benchmark datasets for various metagenomics methods. Their suitability stems from the fact that the number of species that form the metagenomic community is known as well as their relative abundances. The FAMeS datasets have been designed to model real metagenomic communities in terms of complexity and phylogenetic composition.

There are three datasets: simHC, simMC, simLC corresponding to high, medium and low complexity of the metagenomic community, respectively. We first discuss in detail the results of the three methods for simHC, the highest complexity dataset. simHC consists of closely related strains with similar abundances and no dominant species. The lowest abundance is 255 reads out of 118 000 reads. We then summarize the results for the other two mock communities, simLC and simMC, providing more detail in the Supplementary Methods. The bioinformatics processing in this instance consisted of a BLASTn comparison to all NCBI bacterial genomes (Supplementary Methods). The number of genomes mapped, retrieved from the the BLASTn output was ∼2500.

As discussed below, metaMix outperforms Pathoscope and MEGAN in the community profiling task and consequently in the relative abundance estimation (Table 2).

#### 3.1.1 metaMix

To limit the complexity of the fit, we used the two step procedure described in the Methods and fully implemented in metaMix. We first fitted the mixture model with the complete set of 2500 species and a limited run length of 500 iterations. Based on this analysis, we identified 1312 species supported by at least one read and explored this state space. To limit the computational time, we also considered a stronger approximation, including only the 374 potential species supported by at least 10 sequencing reads. Both approaches generated similar results, albeit the more complex one with 1312 potential species required the quadruple of the computation time (12 h instead of 3 h). metaMix identified 116 species, detecting successfully all the members of the metagenomic community. These were detected on the strain level except in four instances where a different strain of the same species, or different species within the same genus was detected. Four species were identified and not in the simulated dataset, hence can be considered as false positives. In order to assess the variability of metaMix results, we ran the analysis 25 times changing the random seed. We report the number of species detected, the sensitivity and specificity as well as relative abundance estimate measure errors, at various posterior probability cutoffs (Table 1). We summarize the resulting community profile based on one of these runs in Supplementary Table S1. 

**Table 1.** simHC community: number of species detected by metaMix as well as sensitivity, specificity, AVGRE, RRMSE for metaMix at various posterior probability cutoffs (default in bold font)

Cutoff	0.9	0.8	0.7	0.6	0.5
**Sensitivity (mean)**	99.82	**99.96**	99.96	100	100
**Sensitivity (SD)**	0.0036	**0.0017**	0.0017	0	0
**Specificity (mean)**	99.86	**99.82**	99.77	99.73	99.70
**Specificity (SD)**	0.0004	**0.0004**	0.0005	0.0003	0.0001
**RRMSE**	16.69	16.85	16.73	17.50	17.48
**AVGRE**	8.20	8.31	8.16	8.60	8.56
**No. Species–median**	115	**116**	117	118	119
**No. Species–SD**	1.2	**0.9**	1.2	0.7	0.3

The results are average values based on 25 runs.

#### 3.1.2 Pathoscope

Pathoscope identified 47 species. Of these 45 are members of the metagenomic community. 42 are the exact same strain, while 3 are either the same species but different strain, or same genus but different species. However it fails to detect 68 species that are actually present in the mixture. Tuning the parameter that enforces the parsimonious results (any thetaPrior > 10), thereby removing the unique read penalty, Pathoscope behaves as a standard mixture model and identifies 165 species (Table 2). With these settings, it identifies all but one members of the community. The organisms are identified at the strain level, except in three instances where it identified different species within the same genus. The major interpretation issue is the presence of a long tail of species (54 species) that are actually not present in the mixture (Supplementary Table S1). Pathoscope produced the results in 1 min. 

**Table 2.** Number of species identified for the FAMeS simLC and simMC datasets, as well as sensitivity, specificity and abundance estimates error measures RRMSE and AVGRE

	metaMix	Pathoscope	MEGAN
	**simHC**		
**Number of species**	116	165	232
**Sensitivity**	99.96	99.1	100
**Specificity**	99.8	97.7	95.0
**RRMSE**	16.9	36.6	35.9
**AVGRE**	8.3	29.7	18
	**simLC**		
**Number of species**	114	147	208
**Sensitivity**	98.8	97.3	100
**Specificity**	99.8	98.4	95.9
**RRMSE**	21.1	185.6	32
**AVGRE**	8.9	53.3	16.1
	**simMC**		
**Number of species**	115	144	208
**Sensitivity**	98.5	98.2	100
**Specificity**	99.8	98.6	95.9
**RRMSE**	29.6	152.7	31.9
**AVGRE**	12.9	49.2	19.3

The metaMix results are based on 25 runs.

#### 3.1.3 MEGAN

MEGAN identified 232 taxa (Supplementary Table S1). It discovered all original species of the community on the strain level, except for nine instances where it identified the LCA. Aside from the lack of strain or species specificity for 8% of the community members, the main issue is the long tail of false positives, i.e low specificity (Table 2). In the species summary provided by MEGAN, there are 119 taxa (species or higher order) which are not actually present, but supported by a sufficient number of reads (default value: 50 reads) for MEGAN to include these in the output. Results were produced in less than a minute. To lower the false positive rate, we also filtered the BLAST results prior to MEGAN analysis, imposing stringent E-value and similarity cutoffs. An E-value < 1 E-10 removed only 9 entries from the results, requiring similarity > 90% removed only 5, while both filters resulted in 208 taxa in the summary results.

#### 3.1.4 Relative abundances

The primary aim for metaMix is to be a diagnostic tool and to answer whether a species is present or absent from the mixture we study. As a secondary aim, we are also interested in estimating accurately the relative abundance of the present organisms. We can assess the abundance estimates produced by the methods by using error measures such as the relative root mean square error, RRMSE and the average relative error, AVGRE (Supplementary Methods for definition). For metaMix, we use the relative abundance estimates from the 25 runs. For all methods, when the exact strain was not identified but the correct species or genus was, we used this abundance. metaMix produces the most accurate abundance estimates and the results are summarized in Table 2.

#### 3.1.5 Importance of read support parameter

We then assessed the importance of the read support parameter r on the output of metaMix. We ran metaMix on the benchmark simHC FAMeS dataset with r = {10, 20, 30, 50} reads, 25 runs for each (Table 3. We observe that as r decreases, a few more related strains from the reference database that are not in the community are retained in the output. As r increases two similar strains are merged into one. 

**Table 3.** simHC FAMeS dataset

Read Support	metaMix	Pathoscope	MEGAN
**50**	114 (0.9)	131	147
**Sensitivity–Specificity**	99.1–99.9	98.2–99.1	100–98.5
**30**	116 (0.95)	131	156
**Sensitivity–Specificity**	99.96–99.8	98.2–99.1	100–98.2
**20**	124 (1.65)	141	166
**Sensitivity–Specificity**	100–99.5	98.2–98.7	100–98
**10**	155 (1.9)	155	188
**Sensitivity–Specificity**	100–98.2	99.1–98.2	100–97.4

Number of species (SD in parenthesis), sensitivity and specificity by metaMix (25 runs), Pathoscope and MEGAN, as a function of the min. number of reads required for each species to appear in the output. metaMix: r = {10, 20, 30, 50} reads, Pathoscope: thetaPrior>7+ post-run threshold = {10, 20, 30, 50} reads, MEGAN: ‘Min Support’ + post-run threshold = {10, 20, 30, 50} reads.

We compared these results with the output of Pathoscope and MEGAN. None of these methods have a read support parameter serving the same purpose as in metaMix, so we tuned the most relevant parameters in these tools. Pathoscope has a thetaPrior parameter that enforces a unique read penalty. This parameter represents the read pseudocounts for the non-unique matches and the default setting is zero which allows for non informative priors. Using the default setting Pathoscope identifies 47 taxa. When thetaP’s value is in (1,7) it identifies 22 taxa, while with thetaP > 7 it identifies 165. With this latter setting which is the one we chose for the comparison, Pathoscope behaves as a standard mixture model.

MEGAN has a ‘Min Support’ parameter which sets a threshold for the number of reads that must be assigned to a taxon so that it appears in the result. Any read assigned to a taxon not having the required support is pushed up the taxonomy until a taxon is found that has sufficient support. We used Min support = {10, 20, 30, 50} reads. The respective number of taxa in the summary files was 250, 243, 236, 232. We then also applied a post-run read count threshold to both methods’ output summary. We set the threshold for 10, 20, 30, 50 reads, respectively, disregarding taxa that have less than that number of reads assigned to them. In all instances metaMix produces a community profile closer to the real one, along with a better balance of sensitivity and specificity compared with the other two methods (Table 3). Pathoscope finds ∼15 more false positives while MEGAN ∼40 more compared with metaMix at the same read support level, except for the lowest r = 10 where metaMix and Pathoscope achieve the same specificity. We report further results in the supplementaty discussion, using different posterior probability cutoffs for the different r settings.

#### 3.1.6 simMC and simLC communities

The FAMeS project includes two additional mock communities that consist of the same 113 species as simHC, but they differ in their relative abundances setup: in simLC there is one dominant species or a few more in simMC. We ran metaMix 25 times for both, changing the random seed. These two datasets turned out to be more challenging for all three methods, missing or merging together some similar related strains. metaMix outperforms Pathoscope and MEGAN in terms of producing a parsimonious community profile and having the best sensitivity and specificity trade-off (Table 2).

The FAMeS datasets are complex and distinct from typical human clinical samples, putting aside gut microbiome analysis. The differences are the large number of organisms, the presence of closely related strains of similar abundances, as well as the lack of viruses. Nevertheless, they are essential datasets to use as benchmark for examining the performance of the methods in a situation of closely related strains in the sample.

### 3.2 Human clinical sample—low viral load

#### 3.2.1 Protein reference database

For the analysis of human clinical samples, we use a custom reference database that combines viral, bacterial, human and mouse RefSeq proteins (Supplementary Methods). To test metaMix in a clinical setting with a low viral load, we used a brain biopsy RNA-Seq dataset from an undiagnosed encephalitis patient (UCL Hospital, data provided as part of a collaboration with Professor Breuer, UCL). Total RNA was purified from the biopsy and polyA RNA was separated for sequencing library preparation. The Illumina MiSeq instrument generated 20 million paired-end reads. We processed the raw data using the bioinformatics pipeline described in Methods section. The processed dataset consisted of ∼75 000 non-host reads and contigs. Based on the BLASTx output there were 1298 potential species.

#### 3.2.2 metaMix

**Fig. 2.**
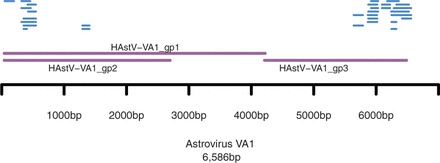
Human clinical sample - novel virus. The reads (short lines) assigned by metaMix to Astrovirus VA1 are aligned to the genome. The longer lines represent the genes of the virus

Following the initial processing, we used metaMix for species identification and abundance estimation. The resulting species profile is shown in Table 4; the 13 metaMix entries correspond to 10 species. The most abundant organism was the ϕX174 bacteriophage, which is routinely used for deep-sequencing quality control. More interestingly, we identified an astrovirus. Five short assembled contigs (44 reads) with length ranging between 167 and 471 bp and two non-assembled reads were assigned to the *Astrovirus VA1* with a probability score of 1 (Fig. 2). metaMix also identified a number of bacteria supported by a few reads. These are either known laboratory reagent contaminants or human skin associated contaminants (Salter *etal.*, 2014). The analysis completed in 29 min. 

**Table 4.** Human clinical sample—novel virus

		metaMix			Pathoscope
Taxon identifier	Scientific name	Assigned reads	Posterior Probability	Bayes factor	Final best hit read numbers
374840	*Enter. phage phiX174*	60447	1	140154	65327
NA	*unknown*	10257	1	NA	NA
9606	*Homo sapiens*	214	1	564	554
28090	*Acinetobacter lwoffii*	94	1	197	126
469	*Acinetobacter*	71	0.99	216	123
13690	*Sphingobium yanoikuyae*	61	0.99	216	135
133448	*Citrobacter youngae*	47	0.91	4	169
645687	*Astrovirus VA1*	46	1	65	46
199310	*Escherichia coli CFT073*	30	1	12	35
56946	*Afipia broomeae*	29	1	29	77
409438	*E.coli SE11*	19	0.98	14	49
618	*Serratia odorifera*	16	0.92	5	—
1747	*Propionibacterium acnes*	13	0.97	14	35
1282	*Staphylococcus epidermidis*	—	—	—	10
28211	*Alphaproteobacteria*	—	—	—	10
28037	*Streptococcus mitis*	—	—	—	8
562	*E.coli*	—	—	—	8
509173	*Acinetobacter baumannii AYE*	—	—	—	7
41297	*Sphingomonadaceae*	—	—	—	6
40214	*Acinetobacter johnsonii*	—	—	—	6
29391	*Gemella morbillorum*	—	—	—	5
76122	*Alloprevotella tannerae*	—	—	—	4
652103	*Rhodopseudomonas palustris*	—	—	—	2
268747	*Prochlorococcus phage*	—	—	—	2

Comparison of community profile: metaMix—pathoscope.

The presence of the astrovirus was confirmed with real-time RT-PCR. Genome sequencing of the astrovirus in the sample and subsequent study of the consensus sequence showed that we had in fact identified a novel virus, closely related to the VA1 strain (Brown *etal.*, 2014).

#### 3.2.3 Pathoscope

Pathoscope identified 22 taxa, corresponding to 15 species and some genera or families (Table 4). It also assigned all 46 reads to the *Astrovirus VA1.* Almost all the species identified from metaMix were identified by Pathoscope, with an additional nine taxa supported by few reads. As the method works only with unassembled sequence data, an extra BLASTx step was performed for the 91 516 reads that had contributed to the 679 assembled contigs. Pathoscope produced the results in  < 1 min.

#### 3.2.4 MEGAN

MEGAN identified 19 taxa and did not detect the astrovirus signal. We modified the minimum read support parameter from 50 reads to 10 to increase sensitivity. MEGAN then identified 25 taxa, including the *Astrovirus VA1.* The remaining 24 were mostly genera, relevant to the species detected by metaMix and Pathoscope. MEGAN produced the results in  < 1 min.

### 3.3 Human clinical sample—species not in the database

We then compared the three methods in a scenario where sequences present in the sample are absent from our reference database. We analyzed 32 million RNA-Seq reads, obtained using the HiSeq instrument for a human clinical sample. Following initial processing using our bionformatics pipeline, the dataset had 1 261 575 non-host sequences for subsequent analyses. There were 3150 potential species based on the BLASTx output.

#### 3.3.1 metaMix

The resulting species profile consisted of 7 species (Supplementary Table S2). The most interesting finding was the identification of *Human coronavirus OC43* (hcov-oc43) with almost a million reads assigned to it. Additionally there were 67 K reads assigned to *Human enteric coronavirus strain 4408.* The presence of both viruses in the results indicated that even though the virus in the sample was mostly similar to hcov-oc 43, there were sequences sharing higher similarity to 4408 at some loci. This is highlighting how the database choice impacts the results: the RefSeq database we used has only one hcov-oc43 strain, while in GenBank there are several, capturing the high mutation rates of this species. We followed up on the sequences assigned to the ‘unknown category’, looking for nucleotide similarity with nr-nt using BLASTn. Half of the reads originated from an untranslated region of the Coronavirus genome, which is not captured by the protein reference database. The remaining reads matched confidently to either zebrafish or chicken sequences, two organisms whose proteins are not in the human microbiome reference we are using. These matches were explained as barcode leakage resulting from multiplexing on the same flowcell zebrafish and chicken RNA-Seq libraries. metaMix appropriately assigned these reads to the ‘unknown’ category, producing a clean probabilistic summary (Supplementary Table S2). The method ran in 4.7 h. The presence of the coronavirus was confirmed using RT-PCR. In this instance, the metaMix results emphasize the importance of being able to deal with missing reference sequences that do not have a closely related strain or species in the same database.

#### 3.3.2 Pathoscope

Pathoscope identified 177 species in this sample. We optimized the value of the unique read penalty parameter and we achieved the best results with the thetaPrior parameter set within the range 10–100. With these settings, the method identified 52 species (Supplementary Table S2). Our assessment is that Pathoscope is confused by the lack of completeness of databases combined with the absence of an ‘unknown’ category, which prevents it from dealing with these unassigned reads sensibly. Pathoscope ran in 10 min.

#### 3.3.3 MEGAN

MEGAN assigned the reads to 30 taxa. These included some species and genera but most were families (Supplementary Table S2). Approximately 250 K reads could not be assigned to any taxonomic level. MEGAN completed its analysis in 8 min.

## 4 Discussion

Here, we present metaMix, a sensitive method for metagenomic species identification and abundance estimation. It is implemented as an R package, freely available from CRAN. Using a Bayesian mixture model framework, we account for model uncertainty by performing model averaging and we resolve ambiguous assignments by considering all reads simultaneously. A key feature of the method is that it provides probabilities that answer pertinent biological questions, in particular the posterior probability for the presence of a species in the mixture. Additionally it accurately quantifies the relative proportions of the organisms.

This general framework is designed to address interpretation issues associated with closely related strains in the sample, low abundance organisms and absence of genomes from the reference database. We show that metaMix outperforms other methods in the community profiling task, particularly when complex structures with closely related strains are studied. As a consequence, it also produces more accurate relative abundance estimates for the species in the mixture. The method can deal with either unassembled reads or assembled contigs or both, allowing for flexibility of choice for the bioinformatics preprocessing. In practice, the choice of bioinformatics processing prior to the application of our Bayesian mixture analysis must be optimized for each application, and our processing pipeline has been designed with viral sequence identification from transcriptome sequencing as a main goal. Nevertheless, as demonstrated by our analysis of the mock bacterial community dataset, the method can be applied in other contexts.

The sensitivity and general applicability of metaMix comes at an increased computational cost, requiring access to a multi-core computer to run efficiently. For the datasets presented here, the computation time remained manageable and did not exceed a few hours, using 12 cores to run 12 parallel chains. Nevertheless, a limitation of metaMix is the increased processing time for very large datasets. Speed related improvements can be implemented in scenarios where the species ambiguity concerns only a small proportion of the read set. Reads with certain assignments can be flagged prior to the MCMC exploration of the state-space. Their assignment information can then be carried forward, thereby reducing the size of the similarity matrix used as input by the mixture model. Another area of possible improvement is MCMC convergence determination. The current version of metaMix produces log-likelihood traceplots allowing the user to visually inspect the MCMC convergence; however, additional diagnostic criteria can be implemented in future versions. Finally, consideration of the differences between mismatches, especially on the amino-acid level could lead to more accurate estimation of the *p*_ij_ probabilities. Such information is captured by the BLASTx similarity score. This metric could thus be used for probability estimation, instead of the number of mismatches.

metaMix is most useful for complex datasets for which the interpretation is challenging. It has been mainly used as a clinical diagnostic tool, helping with the identification of the infecting pathogen while providing an accurate profile of the community in the sample.

## Supplementary Material

Supplementary Data
