# Supplementary material for: Bayesian mixture analysis for metagenomic community profiling
Source: Bioinformatics. 2015 May 21;31(18):2930–8. doi: 10.1093/bioinformatics/btv317 (PMC4565032; doi:10.1093/bioinformatics/btv317)
Supplement: Supplementary Data [file btv317_Supplementary_Data.zip › supplementaryMethods.pdf]

Supporting Information

1 Practical considerations

Rmpi

metaMix uses the Rmpi package (`http://cran.r-project.org/web/packages/Rmpi/`). Rmpi is the R wrapper to MPI, the Message-Passing Interface. MPI is the standardized and portable system allowing parallel programs communicate with each other.

Number of chains and tempering scheme

The optimal number of chains used in the parallel tempering is not obvious. The main idea is that the number of chains used must be large enough to ensure successful swaps between all neighboring chains. The first limitation is the number of chains we can run on a computer. The computing facility we use for all analyses is the UCL CS cluster. The availability of suitable machines as well as considerations towards minimizing the queuing time of the submitted jobs, defines the maximum number we can use as $N = 12$ chains.

The choice of temperature values is motivated by the fact that these must not be too far apart, so that exchange of values between the chains can occur. Additionally the maximum value must be high enough so that no chains become trapped in local minima, hence allowing for global moves.

We implemented a power decay heating scheme:

$$t_n = (t_{n-1} - K)^{\alpha}, \text{ where } n = 2, \ldots, N, \ K\epsilon(0,1) \text{ and } \alpha > 1 \qquad (1)$$

and using $K = 0.001$ and $\alpha = 3/2$ we achieve a slowly heating sequence of chains with a lot of chains similar to the target.

We find that for $N < 10$ the maximum temperature is not very high, hindering a quick global exploration. Ideally we would prefer to run 14 to 20 chains but given our computing constraint $N = 12$ performs satisfactorily.

Number of iterations

Given the described setup of our Parallel Tempering, we find that the MCMC produces reasonable results in (5 $\times$ number of potential species) iterations for each chain. The user can visually inspect the log-likelihood traceplots and assess mixing and convergence of the chain.

Generative probability p_{ij} for unknown category

The generative probability for the "unknown category" is a user-defined parameter and is currently set to be 10^{-6}. This essentialy means that reads with less than $\sim$ 85% similarity to all potential species proteins will be assigned to the unknown category. The default unknown generative probability

for nucleotide comparisons is currently set at 10^{-20}, as it takes into account the genome lengths, as well as the lower level of sequence conservation on the nucleotide level.

2 Parallel Tempering

We use Parallel Tempering MCMC (Earl, 2005) when simple MCMC shows poor mixing, i.e a chain exploring a high probability region may become entraped in it, that is it will find iti difficult to jump to different regions to explore other peaks. A solution is to allow a collection of n parallel Markov Chains exchange information between them. This creates global moves that result in faster mixing.

Each chain simulates from the posterior distribution $P(M_k|X)=g(M_k)$ raised to a temperature $t \in (0,1]$, where model M_k comes from a collection of models $\{M_1, \ldots, M_m\}$. The different temperature levels result in tempered versions of the posterior distribution $P(M_k|X)^{t=1/T}$.

Algorithm 1 Parallel Tempering MCMC algorithm

1. Initialization of Markov chain (done for all n chains).

2. **Mutation step** at iteration t (done for all n chains).

   - Acceptance probability:

     $$A(M_k \rightarrow M_l) = min\{1, \frac{P(X|M_l)^{(t+1)}P(M_l)}{P(X|M_k)^{(t)}P(M_k)}\frac{q(M_l \rightarrow M_k)}{q(M_k \rightarrow M_l)}\} \quad (2)$$

     $q(M_l \rightarrow M_k)$ the probability of transitioning from M_l to M_k.

   - If the step is accepted, the chain moves to proposed state M_l.

   - If not accepted, the chain's current state becomes the previous state of the chain.

3. **Exchange step** when all chains have advanced a prespecified number of iterations, e.g one iteration.

   - Proposes to swap the value of 2 chains adjacent in terms of T, respective chain values M_{k_1} and M_{k_2}, respective temperatures $t_1 = \frac{1}{T_1}$ & $t_2 = \frac{1}{T_2}$, $T_1 < T_2$.

   - Acceptance probability (Jasra 2007):

     $$A = min\{1, \frac{g_{k_1}(M_{k_2})}{g_{k_1}(M_{k_1})}\frac{g_{k_2}(M_{k_1})}{g_{k_2}(M_{k_2})}\} \quad (3)$$

When M_{k_2} has a higher probability than M_{k_1}, the exchange will always be accepted. This is simple to show considering $g_{k_1}(M_{k_1}){=}P(M_{k_1}|X)^{t_1}$ and $g_{k_2}(M_{k_2}) = P(M_{k_2}|X)^{t_2}$:

$$\log \frac{g_{k_1}(M_{k_2})}{g_{k_1}(M_{k_1})} \frac{g_{k_2}(M_{k_1})}{g_{k_2}(M_{k_2})} = \log \frac{P(M_{k_2}|X)^{t_1} P(M_{k_1}|X)^{t_2}}{P(M_{k_1}|X)^{t_1} P(M_{k_2}|X)^{t_2}} \quad (4)$$

$$= (t_1 - t_2)(\log P(M_{k_2}|X) - \log P(M_{k_1}|X)) \quad (5)$$

Since $t_1 > t_2$ and $\log P(M_{k_2}|X) > \log P(M_{k_1}|X)$, the move is always accepted.

3 Relative abundance estimation

We estimate the relative abundances $\boldsymbol{w}$ for a given set of species, using either a frequentist - the Expectation-Maximization algorithm - or a Bayesian approach - the Gibbs Sampler.

Let us recall the metagenomic mixture model notation introduced in the main text. The data consist of N sequencing reads $\boldsymbol{X} = (x_1, \ldots, x_N)$. The relative abundance proportions, that is the proportion of each of the k species in the mixture is denoted by $\boldsymbol{w} = (w_1, ..., w_K)$. The probability of observing the read x_i conditional on the assumption that it originated from species S_j is $p(x_i|x_i \text{ from } S_j) = p_{ij}$.

In the mixture model setting each read x_i, $1 \leq i \leq n$ is assumed to arise from a specific but unknown component of the mixture. The mixture structure is deconvoluted by the introduction of latent variables: we associate x_i with $\boldsymbol{z_i} = (z_{i1}, \ldots, z_{ik})$, a k-dimensional vector indicating to which component x_i belongs.

$$z_{ij} = \begin{cases} 1 & \text{if } x_i \text{ belongs to class j} \\ 0 & \text{otherwise} \end{cases} \quad (6)$$

Expectation Maximization approach

The EM approach to parameter estimation obtains point estimates of the parameters by maximizing the likelihood (Dempster and Laird, 1977).

The algorithm iterations consist of two steps. In the first step, the expected value of the missing variables z_i is computed based on $p(z|x, w)$. In the next step we calculate the new mixing parameters $\boldsymbol{w}$ that maximize the expected complete-data log likelihood

$$\mathbb{E}[\ln p(X, \boldsymbol{z}|\boldsymbol{w})] = \sum_z p(z|x, w) \ln p(x, z|w) \quad (7)$$

with the complete-data likelihood given by

$$p(X, \boldsymbol{z}|\boldsymbol{w}) = \prod_{i=1}^{n} \prod_{j=1}^{k} (w_j p_{ij})^{z_{ij}} \quad (8)$$

Algorithm 2 EM algorithm

- Initialization $\boldsymbol{w}^{(0)}$
- At iteration t

1. **Expectation step.** Generate $z_i^{(t)}$ from $p(z_i^{(t)} = j|x_i, w_j^{(t-1)})$.

$$\hat{z_{ij}} = \frac{p(z_i = j, x_i|\boldsymbol{w})}{p(x_i|\boldsymbol{w})} = \frac{p(z_i = j|\boldsymbol{w})p(x_i|z_i = j, \boldsymbol{w})}{\sum_{j=1}^{k} p(z_i = j|\boldsymbol{w})p(x_i|z_i = j, \boldsymbol{w})} = \frac{w_j p_{ij}}{\sum_{j=1}^{k} w_j p_{ij}} \tag{9}$$

2. **Maximization step.** Given z_i from E-step, calculate new $\boldsymbol{w}^{(t)}$ that maximize the expectation of the complete-data log-likelihood (eq.7).

$$w^{(t)} = \underset{w}{\operatorname{argmax}} \, \mathbb{E}[\ln p(X, \boldsymbol{z}|\boldsymbol{w})] \tag{10}$$

It can be shown that this

$$w^{(t)} = \frac{\sum_{i=1}^{n} z_i^{(t)}}{N} \tag{11}$$

Gibbs sampling approach

The Gibbs sampler is a Markov Chain Monte Carlo method based on the successive simulation of $\boldsymbol{z}$ and $\boldsymbol{w}$ (Diebolt and Robert, 1994). After convergence we obtain the full posterior distribution of $\boldsymbol{w}$. A practical prior for the mixing parameters $\boldsymbol{w}$ is the Dirichlet distribution, owing to its conjugate status to the multinomial distribution.

$$\pi(\boldsymbol{w}) = Dir(\alpha_1, \ldots, \alpha_k) = \frac{\Gamma(\sum\limits_{j=1}^{k} a_j)}{\prod\limits_{j=1}^{k} \Gamma(\alpha_j)} \prod_{j=1}^{k} w_j^{\alpha_j - 1}, \text{ where } \boldsymbol{\alpha} \text{ is positive} \tag{12}$$

Algorithm 3 Gibbs Sampler algorithm

- Initialization $\boldsymbol{w}^{(0)}$
- At iteration t

1. Generate $z_i^{(t)}$ from $p(z_i^{(t)} = j|x_i, w_j^{(t-1)})$. So

$$z_i \sim Mult(1; \hat{z_{i1}}^{(t-1)}, \ldots, \hat{z_{ik}}^{(t-1)}) \tag{13}$$

where $\hat{z_{ij}}$ is given by equation 9.

2. Compute $n_j^{(t)} = \sum_{i=1}^{n} z_{ij}^{(t)}$.

3. Generate $\boldsymbol{w}^{(t)}$ from

$$\pi(\boldsymbol{w}|z^{(t)}) \sim D(a_1 + n_1^{(t)}, \ldots, a_k + n_k^{(t)}) \tag{14}$$

(14) can be explained as $\pi(\boldsymbol{w}|\boldsymbol{z}) \propto \pi(\boldsymbol{z}|\boldsymbol{w})\pi(\boldsymbol{w})$, where for the mixing parameters $\boldsymbol{w}$ a conjugate prior $\pi(\boldsymbol{w})$ is the Dirichlet distribution with parameters $\boldsymbol{\alpha} = \{\alpha_1 \ldots, \alpha_k\}$. Additionally:

$$\pi(\boldsymbol{z}|\boldsymbol{w}) = \prod_{i=1}^{n} \pi(z_i|\boldsymbol{w}) = \prod_{i}^{n} w_1^{z_{i1}} \ldots w_k^{z_{ik}} = \prod_{i=1}^{n} \prod_{j=1}^{k} w_j^{z_{ij}} = \prod_{j=1}^{k} w_j^{n_j} \tag{15}$$

hence sampling $\boldsymbol{w}$ from $Dir \sim (\alpha_1 + n_1, \ldots, \alpha_k + n_k)$.

4 Error measures for abundance estimates

For the FAMeS dataset, we assess the abundance estimates produced by the methods by using error measures such as the relative root mean square error, RRMSE and the average relative error, AVGRE.

$$\textbf{RRMSE} = \sqrt{\frac{1}{K} \sum_{j=1}^{K} (\frac{|w_j - t_j|}{t_j})^2} \tag{16}$$

$$\textbf{AVGRE} = \frac{1}{K} \sum_{j=1}^{K} (\frac{|w_j - t_j|}{t_j}) \tag{17}$$

where t_j is the true abundance of species j and w_j the estimated abundance.

5 Importance Sampling, Defensive Sampling, MLE approximation

Accounting for the uncertainty in mixture weights $\boldsymbol{w}$, a Monte Carlo approximation can be used for the marginal likelihood, by drawing independent samples from the prior to estimate $P(X)$ and averaging the likelihood. The simulation from the prior is computationally inefficient, as the majority of samples are outside the regions of high likelihood. Importance sampling (IS) techniques can be used to reduce the variance of the estimator. To obtain an efficient IS proposal distribution, we first approximate the posterior distribution $P(\boldsymbol{w}|X)$ with a normal multivariate distribution g, based on the samples generated by the Gibbs sampler. We then generate n samples $\boldsymbol{y_i} \sim \mathcal{N}(\boldsymbol{\mu}, \boldsymbol{\Sigma})$, $1 \leq i \leq n$. Defining the IS weights $a_i = \frac{\pi(\boldsymbol{y_i})}{g(\boldsymbol{y_i})}$, the IS estimator of the marginal likelihood is then:

$$\hat{I} = \frac{\sum_i a_i P(X|y)}{\sum_i a_i} \tag{18}$$

The method works well if the importance distribution is fairly similar to the target distribution but with heavier tails. Using the posterior approximation as the importance distribution means that the IS estimator becomes the harmonic mean estimator (HME). HME is known to be unstable and to overestimate the marginal likelihood $P(X)$. In order to overcome this issue and to make the distribution tails heavier, we perform defensive importance sampling by using a mixture of posterior and prior as the IS distribution (Hesterberg, 1995). This approach is only slightly costlier in computational time compared to the typical IS.

As discussed in the main text, for the IS we have approximated the posterior distribution $P(\boldsymbol{w}|X)$ with a normal multivariate distribution g, based on the output of the Gibbs sampler. However the posterior approximation g as IS proposal distribution means that the marginal likelihood estimator becomes the Harmonic Mean estimator. This estimator can be unstable; a solution to this problem is the defensive importance sampling (Hesterberg, 1995). The main idea is the incorporation of a heavy tail component in the importance function g , effectively substituting it by the mixture density:

$$\lambda g(y) + (1 - \lambda) q(y), 0 < \lambda < 1 \tag{19}$$

where λ is close to 1. A natural choice for q as the stabilizing factor is the prior density π.

In practice that means that the samples we use for the defensive IS estimator are generated with probability λ from g and with probability $1 - \lambda$ from π .

Comparison of the 3 approaches

We compared the performance of metaMix on the same simHC FAMeS dataset, using Importance Sampling and Defensive Importance Sampling (95% samples

produced by posterior approximating g and 5% by π) for the marginal likelihood estimation as well as using the MLE approximation. The latter are the results presented in the main text. For the IS and the defensive IS at each MCMC iteration 1,000 samples were drawn from the proposal distribution.

The resulting species profiles can be seen in table S3. We ran the MCMC for 1,000 iterations in order to obtain results within 24 hours. All three approaches produced almost identical results, in terms of species identified and abundance estimates, with the defensive IS performing a bit better in terms of abundance estimation accuracy. However the MLE approximation method was ~13x times faster than the other two, reducing the time required from ~19 hours to 90 minutes.

Table 1: FAMeS simHC - comparing the effect of different marginal likelihood estimation methods on metaMix performance: species profiling, accuracy of abundance estimation and computational time.

	**Importance Sampling**	**Defensive Sampling**	**MLE approximation**
Number of species	116	116	116
$\boldsymbol{w}$ estimate - rRMSE	17	16.8	17.1
$\boldsymbol{w}$ estimate - AVGRE	8.5	8.3	8.6
Computational time (hours)	18.6	18.6	1.5

6 simHC - assembled data

The results we have reported in the main text far are based on unassembled simHC FAMeS data. We subsequently wanted to compare the performance of metaMix on the same dataset, doing first an assembly step. We used Velvet with a high kmer value ($k = 89$) in order to obtain high quality contigs. This resulted in 733 contigs made up by 2,403 reads, i.e approximately 2% of the total reads were contributing to contigs. We then annotated contigs and unassembled reads with BLASTn and applied metaMix with default parameters. We find all members of the metagenomic community and one false positive (based on one run: sensitivity=100, specificity=99.96). The estimates for relative abundance were also close to the true values ($RRMSE = 16.9$ and $AVGRE = 8.2$). We therefore observe that the metaMix results are very similar whether we choose to include or forego an assembly step, with the resulting community profile very close to the true one.

7 simMC, simLC - additional simulations

For the simMC and simLC communities we also ran metaMix for different posterior probability cutoffs (0.5-0.9) and different read support values ($\mathbf{r}$={10,20,30}. We present the results in Tables 2 and 3. Naturally, as we allow species with

lower posterior probabilities in the results, the sensitivity increases and the specificity decreases. Changing the read support value and comparing with MEGAN and Pathoscope, we observe the same pattern as for simHC: metaMix has the best balance of specificity and sensitivity between the three methods.

Table 2: simLC, simMC: Number of species detected by metaMix as well as sensitivity, specificity, AVGRE, RRMSE for metaMix at various posterior probability cutoffs. The results are average values based on 25 runs.

**Cutoff**	**0.9**	**0.8**	**0.7**	**0.6**	**0.5**
			**simLC**		
**Sensitivity (mean)**	98.32	**98.82**	99.00	99.07	99.11
**Sensitivity (sd)**	0.0083	**0.0050**	0.0030	0.0018	0
**Specificity (mean)**	99.89	**99.85**	99.82	99.78	99.75
**Specificity (sd)**	0.0004	**0.0003**	0.0004	0.0004	0.0002
**# Species - median**	113	**114**	115	116	117
**# Species - sd**	1.4	**1.1**	1.0	0.9	0.4
**rRMSE**	21.1	**21.0**	21.1	21.3	21.6
**AVGRE**	8.9	**8.8**	8.9	8.9	9.2
			**simMC**		
**Sensitivity (mean)**	97.96	**98.46**	98.79	98.93	99.11
**Sensitivity (sd)**	0.0061	**0.0048**	0.0044	0.0036	0
**Specificity (mean)**	99.83	**99.77**	99.71	99.66	99.63
**Specificity (sd)**	0.0005	**0.0004**	0.0004	0.0004	0.0002
**# Species - median**	114	**115**	118	119	120
**# Species - s.d**	1.17	**1.07**	1.08	1.08	0.48
**RRMSE**	29.98	**29.93**	30.05	30.11	29.96
**AVGRE**	13.05	**13.18**	13.31	13.37	13.26

Table 3: simLC, simMC FAMeS datasets: number of species detected as well as sensitivity and specificity of metaMix, Pathoscope and MEGAN, as a function of the minimum number of reads required for each species to appear in the output. For metaMix that is r={10, 20, 30} reads, for Pathoscope thetaPrior> 7+ post-run threshold ={10, 20, 30} reads, for MEGAN ”Min Support” + post-run threshold ={10, 20, 30} reads.

	**metaMix**	**Pathoscope**	**MEGAN**
		**simLC**	
**r30**	114 (1.09)	126	142
**Sensitivity- Specificity**	98.82 99.84	97.32 - 99.27	100 - 98.71
**r20**	116 (1.17)	127	147
**Sensitivity- Specificity**	98.89 99.76	97.32 - 99.22	100 - 98.5
**r10**	133 (1.22)	131	157
**Sensitivity- Specificity**	100 99.11	97.3 - 99.05	100 - 98
		**simMC**	
**r30**	115 (0.69)	126	141
**Sensitivity- Specificity**	98.46 99.77	98.21 - 99.35	99.1 - 98.8
**r20**	117 (1.1)	126	145
**Sensitivity- Specificity**	98.21 99.67	98.21 - 99.35	99.1 - 98.6
**r10**	144 (2.3)	130	158
**Sensitivity- Specificity**	99.46 98.56	98.2 - 99.18	99.1 - 98

Finally we calculate separately RRMSE for rare and abundant species 4. We define as abundant the species that have a relative abundance greater than 10%. Using this criterion, in simLC there is one such species while in simMC there are two. The metaMix results are based on 25 runs.

Table 4: simLC, simMC FAMeS dataset: combined and separate RRMSE calculations.

	**metaMix**	**Pathoscope**	**MEGAN**
		**simLC**	
**RRMSE.all**	21.1	185.6	32.0
**RRMSE.abundant**	2.0	41.6	3.1
**RRMSE.rare**	21.2	186.4	32.1
		**simMC**	
**RRMSE.all**	29.6	152.7	31.9
**RRMSE.abundant**	5.4	28.6	8.7
**RRMSE.rare**	29.9	154.1	32.1

8 Reference databases

8.1 Reference database for FAMeS datasets

As mentioned in the main text, all NCBI bacterial genomes (`ftp://ftp.ncbi.nlm.nih.gov/genomes/Bacteria/all.fna.tar.gz`) were used to construct the database. Additionally, there were six organisms in the dataset that were missing from the database, so we added these manually (taxon identifiers: 155919, 155920, 165597, 286604, 321955, 332415).

8.2 Protein reference database for human clinical samples

For the analysis of human clinical samples, we created a custom reference database consisting of viral, bacterial, human and mouse RefSeq proteins. More specifically, all viruses are used `ftp://ftp.ncbi.nlm.nih.gov/refseq/release/viral/viral.1.protein.faa.gz`, as well as all the bacteria of the human microbiome, according to `ftp://ftp.ncbi.nih.gov/genomes/HUMAN_MICROBIOM/Bacteria/all.faa.tar.gz`.
